# Crystal structures of *Mycobacterium tuberculosis* and *Mycobacterium thermoresistibile* glycyl-tRNA synthetases in various liganded states

**DOI:** 10.1371/journal.pone.0326500

**Published:** 2025-06-30

**Authors:** Michael K. Fenwick, Amy E. DeRocher, Justin K. Craig, Elizabeth K. Harmon, Steve Seibold, Lijun Liu, Kevin P. Battaile, Lynn K. Barrett, Wesley C. Van Voorhis, Isabelle Q. Phan, Bart L. Staker, Sandhya Subramanian, Scott Lovell, Peter J. Myler

**Affiliations:** 1 Seattle Structural Genomics Center for Infectious Disease, Seattle, Washington, United States of America; 2 Center for Global Infectious Disease Research, Seattle Children’s Research Institute, Seattle, Washington, United States of America; 3 Department of Medicine, Division of Allergy and Infectious Diseases, Center for Emerging and Re‐emerging Infectious Diseases (CERID), University of Washington, Seattle, Washington, United States of America; 4 Protein Structure and X-ray Crystallography Laboratory, The University of Kansas, Lawrence, Kansas, United States of America; 5 New York Structural Biology Center, New York, New York, United States of America; 6 Department of Biomedical Informatics and Medical Education, University of Washington, Seattle, Washington, United States of America; 7 Department of Global Health, University of Washington, Seattle, Washington, United States of America; University of Delhi, INDIA

## Abstract

Glycyl tRNA synthetases (GlyRSs) are prospective drug targets for combating *Mycobacterium tuberculosis* (*Mtb*) and cancer in humans. These synthetases are of the α2-subtype, with the ortholog in humans being dual targeted to the cytosol and mitochondria. Whereas the human enzyme has been structurally characterized previously in several liganded states, no structures of *Mtb*GlyRS have thus far been reported. Here, we describe our recent work with *Mtb*GlyRS and the closely-related *Mycobacterium thermoresitibile* GlyRS (*Mtr*GlyRS), which progressed through all phases of the structural genomics pipeline, for the purpose of facilitating structure-based drug discovery. *Mtb*GlyRS was expressed in *Mycobacterium smegmatis* and *Mtr*GlyRS in *Escherichia coli*. Crystal structures are described for complexes of the two enzymes with adenosine monophosphate (AMP) and glycyl-sulfamoyl-adenylate (glycyl-AMS) at resolutions of 1.65/2.90 and 2.25/1.95 Å, respectively, and for *Mtr*GlyRS in its apo state at 2.85 Å. Despite crystallizing in the dimeric state characteristic of many class II synthetases, the two enzymes elute predominantly as monomers during size exclusion chromatography. Strikingly, significant portions of the dimer interface and active site are unstructured in the *Mtr*GlyRS apoenzyme crystal. AMP orders two tRNA recognition loops and a section of the insertion domain, and glycyl-AMS further stabilizes the structure, including the closure of a lid motif. Both the active and anticodon binding sites display structural differences with the human GlyRS and thus the collection of crystal structures should be useful for guiding drug development efforts targeting the various characterized structural states.

## Introduction

Tuberculosis continues to be a tremendous health and economic burden, with greater than 10 million incidences, 1.2 million deaths, and the equivalent of 6 billion US dollars globally budgeted annually [1,2]. It is estimated that two billion people have latent tuberculosis [3]. Unfortunately, treatment schedules range from six months to two years depending on the degree of evolved resistance and latency, with an alarmingly high percentage of patients not completing treatment [4]. There has also been increased incidences of pulmonary infections and lung diseases associated with non-tuberculosis *Mycobacteria* such as the *Mycobacterium avium* complex, *Mycobacterium abscessus*, and *Mycobacterium kansasii* in both the US and worldwide, with estimated 5 year mortality rates of nearly 25% [5,6]. From the standpoint of structural genomics, these challenges urge the characterization of additional molecular targets that have druggable cavities and display sufficient structural differences with their human counterparts to minimize risks of off target binding and toxicity.

The structures of aminoacyl tRNA synthetases (AaRSs) typically meet these criteria [7–10], and there are currently two FDA approved drugs targeting AaRSs, mupirocin and tavaborole, which inhibit IleRS and LeuRS, respectively [11–15]. Their prospects for druggability are further underscored by the large number of natural product inhibitors identified thus far against synthetases from many subclasses [16–18]. Known synthetic inhibitors developed against the *M. tuberculosis* AaRSs target LeuRS, MetRS, TyrRS, AspRS, LysRS, or PheRS [19–26], with Epetraborole exhibiting broad-spectrum potential [27, 28].

Structurally, the AaRSs comprise two enzyme classes that are subdivided into six or seven subclasses [Ia-c and IIa-c(d)] based on the sequences and structures of their catalytic domains [29–32]. While the majority of class II synthetases form α_2_ homodimers, the member glycyl-tRNA synthetases (GlyRSs) exist as either α_2_ homodimers, (αβ)_2_ heterotetramers, or (α − β)_2_ homodimers [33–35]. Structures of all three forms – complete, or in some cases partial – have now been determined using X-ray diffraction or cryo-EM [32, 36–41]. Both Mycobacterial and human cells express the α_2_ subtype, and notably, the GlyRS gene in humans encodes one product that serves both the cytosol and mitochondria [42–44], which has obvious advantages for the design of selective inhibitors. Whereas the human GlyRS (*Hs*GlyRS) has been structurally characterized in various mechanistically meaningful states, including complexes with tRNA [37, 38, 45, 46], Mycobacterial GlyRSs and other α2-type GlyRSs from Gram positive bacteria have not yet been structurally described.

The GlyRSs expressed by several types of *Mycobacteria* have recently been entered into the SSGCID pipeline. Notably, in addition to being an essential gene, *MtbglyS*/*glyQS* was shown previously to be the most vulnerable among those encoding AaRSs in *M. tuberculosis* strain H37Rv [47], which provided a strategic basis for target selection. Additionally, recent pipeline work has demonstrated that both the *M. tuberculosis* GlyRS (*Mtb*GlyRS) and *Mycobacterium thermoresistibile* GlyRS (*Mtr*GlyRS) are tractable platforms for enzyme expression, crystallization, and structure-based drug design. Methodologies are also available in the literature for purification and crystallization of *Hs*GlyRS [37, 38, 48, 49].

A commonly employed strategy for AaRS inhibition is to derivatize the aminoacyl-adenylate intermediate. Two inhibitors that closely resemble the intermediate are ascamycin, a structural analog of alanyl-adenylate [50], and the processed *C*-terminus of microcin C, an analog of aspartyl-adenylate [51]. Both have been derivatized into their respective aminoacyl-sulfamoyl-adenylates (aminoacyl-AMSs) [51, 52], which comprise a larger class of compounds known to be highly potent inhibitors of many AaRSs [53–56]. Expectedly, aminoacyl-AMSs have been shown in crystal structures to occupy both the cognate L-amino acid and AMP binding sites, and typically stabilize more ordered active site conformations [57, 58]. The ability to order the catalytic domains also makes them attractive compounds for determining AaRS crystal structures within structural genomics pipelines. Moreover, inhibitor platforms have been developed for targeting the human GlyRS based on glycyl-AMS [59–61].

In the present study, high-resolution crystal structures of *Mtb*GlyRS and *Mtr*GlyRS bound to glycyl-AMS were determined by X-ray diffraction to resolutions of 2.25 and 1.95 Å, respectively. Both proteins were produced in milligram amounts, although obtaining the *M. tuberculosis* form was enabled by expression in *Mycobacterium smegmatis*. In contrast to previously solved structures containing related ligands [45, 62], the structures presented here also detail the magnesium coordination sphere. Crystal structures were also obtained for the AMP-bound states of the two orthologs, and in the case of *Mtr*GlyRS, the apo state. The ensemble of structures provides a clearer description of the active sites given differences in the crystallization conditions. Below, we characterize the two Mycobacterial GlyRSs, including their oligomeric states during purification, the AMP and glycyl-AMS binding sites, and ligand-dependent conformational changes and dynamics in the active site and dimer interface.

## Materials and methods

### Cloning, and protein expression and purification

*Mtb*GlyRS was insoluble when expressed in *E. coli;* therefore, we used *M. smegmatis* as a protein expression system. Plasmid pDTCF containing the MycORI origin of replication [63] was provided as a gift by Dr. Vishant Boradia (Seattle Children’s Research Institute, Center for Global Infectious Disease Research). *Mtb*GlyRS with an MHHHHHHHHGGGG tag at the *N*-terminus was subcloned into pDTCF using NEB builder, and *M. smegmatis* strain MC2_155 was transformed with the plasmid under selection with hygromycin [64]. For large-scale cultures, 4 l of *M. smegmatis* were grown at 37°C in 7H9 broth under selection with hygromycin. When the optical density at 600 nm reached 0.4, 100 ng/ml anhydrotetracycline was added to induce protein expression. Cells were harvested by centrifugation after 20 h yielding 31 g of pellets (wet weight), flash frozen in dry ice ethanol, and stored at −80°C.

To optimize *Mtb*GlyRS purification, several lysis and purification conditions were tested, including varying pH, buffer identity, ionic strength, monovalent cation identity, Mg^2+^ concentration, presence or absence of detergent, and presence or absence of ATP. The cell pellet was suspended in 150 ml buffer A (20 mM Tris HCl, pH 8.2, 300 mM KCl, 10 mM MgCl_2_, and 5% glycerol), supplemented with 4 mM disodium ATP (VWR) and 2 EDTA-free cOmplete protease inhibitor tablets (Roche Diagnostics), and lysed by sonication using a Branson sonifier fitted with a flat tip (70% power for 10 minutes, with 5 second pulses separated by 10 second pauses). The lysate was treated with 2 µL Benzonase® nuclease (25 U/µl, Avantor) for 40 minutes then clarified by centrifugation at 10,000 x g for 40 minutes. The supernatant was loaded onto a 5 ml HisTrap Fast Flow column (Cytiva) equilibrated with buffer A containing 1 mM ATP and 20 mM imidazole, washed, and eluted with a linear gradient of 20–500 mM imidazole in buffer A. Fractions containing GlyRS were loaded onto a HiLoad Superdex 75 26/600 column (Cytiva) equilibrated with buffer A containing 1 mM ATP. The purified protein was then concentrated to 14.8 mg/ml based on the absorbance at 280 nm, flash frozen in liquid nitrogen, and stored at −80°C.

*Mtr*GlyRS was prepared according to the SSGCID “tier 1” procedures [65]. The protein was expressed in 2 l cultures of *E. coli* Rosetta (DE3) R3 cells from plasmid pAVA0421 [65] using autoinduction [66, 67] to yield the following product: MAHHHHHHMGTLEAQTQGPGSM_1_…C_461_. Frozen bacterial cell pellets were thawed by vortexing in 200 ml of lysis buffer (20 mM HEPES, pH 7.0, 300 mM NaCl, 5% glycerol, 30 mM imidazole, 0.5% CHAPS, 21 mM MgCl_2_, and 1 mM TCEP). The cell suspension was sonicated while on ice for 15 minutes (70% amplitude, 5 second ON/10 second OFF cycles) using a Branson 450D Sonifier (Branson Ultrasonics). The sonified solution was then supplemented with 20 µl Benzonase® nuclease (EMD Chemicals) and incubated for 40 minutes at room temperature under gentle agitation. The lysate was clarified by centrifugation at 10,000 rpm for 60 min at 4°C in a F14S Rotor (Thermo Fisher).

The supernatant of the spun lysate was filtered through a 0.45 µm cellulose acetate filter (Corning Life Sciences) and loaded onto a 5 ml HisTrap Fast Flow Ni^2+^-affinity column (Cytiva) pre-equilibrated with column buffer A (20 mM HEPES, pH 7.0, 300 mM NaCl, 5% glycerol, and 30 mM imidazole). The protein was eluted with a linear gradient using a buffer comprising the buffer A components and 500 mM imidazole. The eluted sample was concentrated in an Amicon Ultra Centrifugal Filter device (Millipore Sigma) to less than 5 ml and then fractionated on a HiLoad 26/600 Superdex 200 column (Cytiva) pre-equilibrated with gel filtration buffer (20 mM HEPES, pH 7.0, 300 mM NaCl, 5% glycerol, and 1 mM TCEP). The protein purity in the peak fractions was verified by SDS-PAGE, and the purified protein was concentrated to 17 mg/mL based on the absorbance at 280 nm, flash-frozen in liquid nitrogen, and stored at −80 °C.

### Crystallization and structure determination

All crystallization experiments were set up using an NT8 drop setting robot (Formulatrix, Inc.) and UVXPO MRC (Molecular Dimensions) 96-well sitting drop vapor diffusion plates at 291 K. Glycyl-AMS was purchased from Biosynth and AMP and ATP from Sigma. 100 nL of protein and 100 nL reservoir solution were dispensed and equilibrated against 50 µL of the latter. Crystals were grown and cryoprotected under the following conditions: glycyl-AMS-bound *M. thermoresistibile* GlyRS (2 mM glycyl-AMS added prior to crystallization), reservoir, Hampton Index condition F11 (0.2 M NaCl, 0.1 M Bis-Tris, pH 6.5, and 25% PEG 3350), cryoprotectant, 85% reservoir/15% glycerol; apo and AMP-bound *M. thermoresistibile* GlyRS (both supplemented with 2 mM AMP), reservoir, Rigaku JCSG+ condition H8 (25% PEG 3350, 0.1 M Bis-Tris, pH 5.5, and 0.2 M NaCl), cryoprotectant, 85% reservoir/15% PEG 200; glycyl-AMS- (crystals soaked 4 hours in crystallant containing 5 mM glycyl-AMS) and AMP-bound *M. tuberculosis* GlyRS (protein samples contained ATP and MgCl_2_), reservoir, Molecular Dimensions Morpheus Screen condition A2 (20% ethylene glycol, 10% PEG 8000, 100 mM imidazole/MES, pH 6.5, 30 mM MgCl_2_, and 30 mM CaCl_2_), cryoprotectant, reservoir (fresh drop).

X-ray diffraction data were collected at the National Synchrotron Light Source II beamline 19-ID (NYX) using an Eiger2 XE 9M pixel array detector. Intensities were integrated using XDS [68] via AUTOPROC [69] and the Laue class analysis and data scaling were performed with Aimless [70]. Structure solution of *Mtr*GlyRS was conducted by molecular replacement with Phaser [71] using an AlphaFold structure of *Mtr*GlyRS (entry AF-G7CIG9-F1 [72]) as the search model. The refined structure was used for subsequent phasing of the *Mtb*GlyRS diffraction data. Manual model building and automated refinement were performed with the aid of Coot [73] and Phenix [74], respectively, and structural validation with MolProbity [75]*.* The protein assemblies were analyzed with PISA (web, https://www.ebi.ac.uk/pdbe/pisa/, and CCP4i versions [76, 77]). Multiple sequence alignments were performed using Clustal Omega and Clustal X, the latter for its implementation of Q-scores [78–80]. Figures were prepared using Chimera [81, 82], ESPript 3.0 [83], and ChemDraw.

The structures of *Mtb*GlyRS with AMP or glycyl-AMS belong to space group *P*6_1_22 with one protein chain in the asymmetric unit (asu), have similar unit cell constants, and were refined at 1.65 and 2.25 Å, respectively. The corresponding *Mtr*GlyRS structures belong to space group *C*2 with two proteins in the asu and have resolutions of 2.90 and 1.95 Å. The *Mtr*GlyRS apoenzyme crystals grew in the space group *I*4_1_32 with one copy of the protein in the asu and diffracted X-rays to 2.85 Å. Additional diffraction and refinement data are provided in Table 1.

**Table 1:** X-ray diffraction and structure refinement statistics.

	*Mtb*GlyRS *Glycyl-AMS*	*Mtb*GlyRS *AMP*	*Mtr*GlyRS	*Mtr*GlyRS *Glycyl-AMS*	*Mtr*GlyRS *AMP*
**Accession codes**					
SSGCID	MytuD.19107.a	MytuD.19107.a	MythA.19107.a	MythA.19107.a	MythA.19107.a
PDB	8U2P	8T5N	8SLD	8SLG	8SLF
**X-ray diffraction data**					
wavelength (Å)	0.9795	0.9795	0.9795	0.9795	0.9795
space group	*P*6_1_22	*P*6_1_22	*I*4_1_32	*C*2	*C*2
*Unit cell*					
*a, b*, *c* (Å)	124.3, 124.3, 131.4	126.4, 126.4, 129.0	190.8, 190.8, 190.8	170.2, 87.1, 99.4	173.8, 86.3, 99.0
α, β, γ (°)	90, 90, 120	90, 90, 120	90, 90, 90	90, 104.2, 90	90, 104.2, 90
number of reflections	29,045	73,239	14,177	102,442	31,541
redundancy	30.7	35.1	80.2 (85.2)	6.9 (6.6)	3.5 (3.7)
resolution (Å)	49.8–2.25 (2.32–2.25)*	45.14–1.65 (1.68–1.65)	47.71–2.85 (3.00–2.85)	96.32–1.95 (2.00–1.95)	47.98–2.90 (3.06–2.90)
completeness (%)	100.0 (100.0)	100.0 (100.0)	100.0 (100.0)	99.9 (99.9)	99.6 (99.8)
*I*/σ(*I*)	18.1	19.4	30.8	13.0	9.0
*R*_pim_	0.036 (0.439)	0.021 (0.417)	0.02 (0.402)	0.029 (0.453)	0.04 (0.305)
*CC*_1/2_	0.999 (0.718)	1.000 (0.886)	1.000 (0.784)	0.999 (0.829)	0.998 (0.912)
**Structural refinement**					
number of reflections	28,975	73,150	14,147	102,260	31,468
resolution (Å)	45.15–2.25	40.03–1.65	44.98–2.85	48.16–1.95	42.6–2.9
*R*_work_/*R*_free_	0.179/0.208	0.156/0.171	0.233/0.265	0.174/0.201	0.183/0.224
RMSD, bonds (Å)	0.003	0.009	0.002	0.007	0.005
RMSD, angles (°)	0.593	0.976	0.435	0.942	0.744
**Structure validation**					
*MolProbity*					
clash score, all atoms	1.71	1.69	3.2	2.15	3.2
MolProbity score	1.23	0.92	1.36	0.99	1.18
Ramachandran plot					
favored (%)	97.72	98.21	96.28	98.49	97.66
outliers (%)	0.00	0.00	0.00	0.35	0.12

It should be noted that the crystal of apo *Mtr*GlyRS originated from drops containing AMP. Additionally, the AMP-bound *Mtb*GlyRS crystal contains two divalent metal ions in the active site that likely arose from the high ion concentrations in the crystallant (30 mM MgCl_2_ and 30 mM CaCl_2_). One of the ions alters the conformations of Arg291 and Glu165. The second is ligated by Glu284,a ribose binding residue, and its coordination complex is associated with a noticeable conformational change at His256. On the other hand, the *Mtb*GlyRS-glycyl-AMS complex contains a single divalent cation coordinated by the sulfamoyl group and Glu284 carboxylate. Its structural relevance is supported by its close resemblance to related divalent metal coordination spheres observed in other class II AaRSs (see, e.g., Refs. [84, 85]). Waters 603A and 604B in the structure of glycyl-AMS-bound *Mtr*GlyRS are likely divalent metals; the equivalent *Mtb*GlyRS structure models a metal explicitly. Collectively, the two sets of structures aid the interpretation of the active site architectures.

## Results

### Expression and chromatographic profiling of Mycobacterial GlyRSs

*Mtb*GlyRS and *Mtr*GlyRS are encoded by the *glyQS* gene and are 463 and 461 residues in length, with theoretical molecular weights of 52.9 and 52.7 kDa, respectively. The two synthetases are 79.8% identical in their amino acid sequences [86], but phylogenetically branch within separate clades when aligned with eleven other Mycobacterial GlyRS sequences (S1 Fig in S1 File). The two groups of synthetases utilize different insertion domains, with the group containing *Mtr*GlyRS having a zinc binding motif.

**Fig 1:**
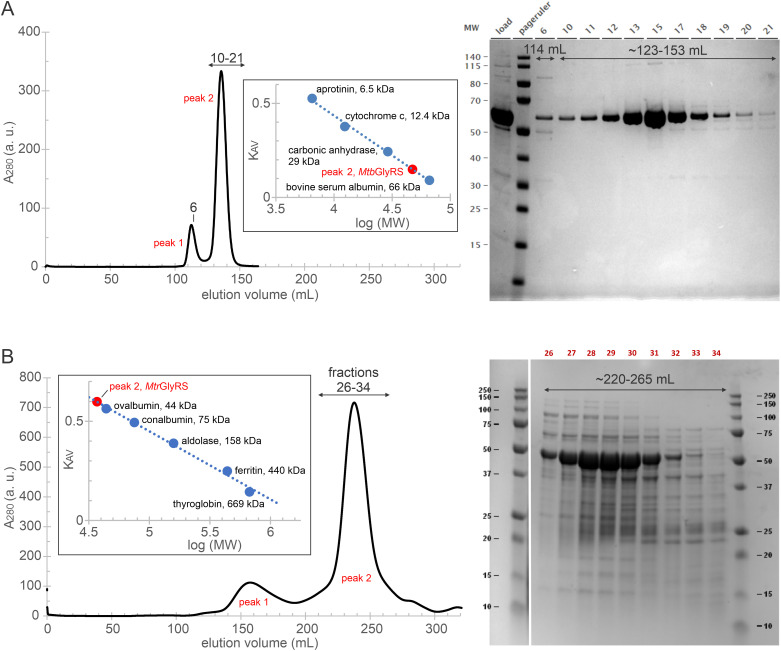
*Mtb*GlyRS and *Mtr*GlyRS purify predominantly as monomers. **(A)** *Mtb*GlyRS purified using a HiLoad 26/600 Superdex 75 column runs mostly at 47.3 kDa. **(B)** *Mtr*GlyRS purified using a HiLoad 26/600 Superdex 200 column elutes with an apparent mass of 36.9 kDa.

*Mtb*GlyRS and *Mtr*GlyRS were both successfully purified, albeit using different methodologies [65], resulting in several PDB entries. Table 1 summarizes a subset of five structures analyzed here. *Mtr*GlyRS was readily produced using standard protocols, and 37.3 mg of protein were purified from 2 l of culture. In contrast, *Mtb*GlyRS was insoluble when expressed in *E. coli*. GlyRSs from several *Mycobacterium* species – *M. abscessus, M. avium, M. fortuitum, M. bortonae, M. marinum* and *M. smegmatis* – were also insoluble when expressed in *E. coli*. To produce *Mtb*GlyRS, the *glyQS* gene (with an *N*-terminal purification tag) was cloned into an *M. smegmatis* expression vector pDTCF and transformed into *M. smegmatis*, and expression of *Mtb*GlyRS was induced by anhydrotetracycline. Standard lysis and purification buffers were modified to improve solubility and yield. Six milligrams of the protein were purified from 4 l of culture. Notably, both proteins eluted predominantly as monomers from size exclusion chromatography columns (Fig 1), which was unanticipated given their expected dimeric functional state and previous chromatographic results for other GlyRS orthologs [33, 87, 88].

### Subunit architecture and conserved motifs

**Fig 2:**
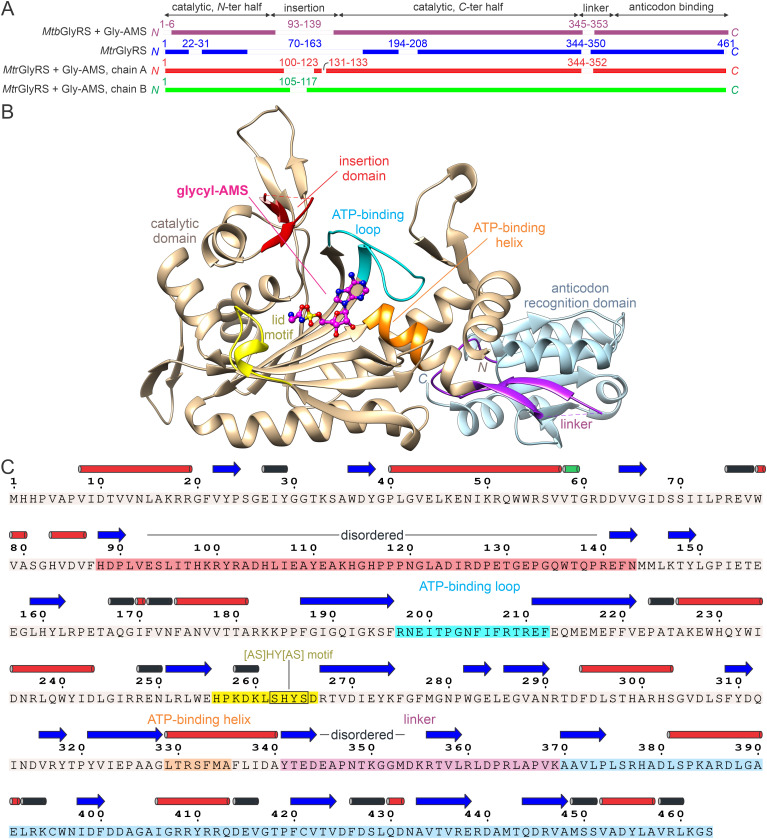
Subunit architecture. **(A)** Amino acid residues modelled into electron density maps. Ranges of unstructured residues are listed above the sequences. **(B)** Structural domains and glycyl-AMS binding elements in *Mtb*GlyRS. Catalytic domain, residues 1-87 and 144-340, tan; insertion domain, residues 88-143, red; β-hairpin linker, residues 341-369, purple; anticodon recognition domain, residues 370-463, light blue; class II signature ATP binding loop, residues 197-212, cyan, and helix, residues 329-335, orange; molecular lid, residues 256-266, yellow. **(C)** Primary and secondary structures. Amino acids are colored as in panel B; secondary structural assignments [82] are shown above the sequence: β-strands, blue arrows; α-, 3_10_, and π helices, red, black, and green cylinders, respectively.

Structures of *Mtb*GlyRS bound to AMP or glycyl-AMS were compared with the corresponding *Mtr*GlyRS complexes as well as with the apo form of *Mtr*GlyRS. The liganded chains are generally much more ordered than the *Mtr*GlyRS apoenzyme, although major sections of the insertion domain are not visible in the various electron density maps (Fig 2A). A few of the modelled *Mtr*GlyRS chains are more complete, however, owing to the presence and stability of the zinc binding site. Electron density maps validating the modeling of ligands and metal coordination spheres in the *Mtb*GlyRS complexes are shown in S2 Fig in S1 File.

*Mtb*GlyRS and *Mtr*GlyRS are the smallest of the α2-type GlyRSs thus far characterized structurally. Alignments of the subunits using DALI show that the closest structural ortholog in the PDB is* Thermus thermophilus* GlyRS (Z-score of 48.3 [89]; PDB entry 1GGM [62]), which contains insertions in each of its three domains. Similar to the *Tt*GlyRS, the two Mycobacterial GlyRS subunits adopt the canonical architecture consisting of an *N*-terminal catalytic domain and a *C*-terminal anticodon binding domain connected by a β-hairpin insertion (Fig 2B and C). The catalytic core contains the class-II specific antiparallel β-sheet having the characteristic insertion domain after the first strand of the core β-sheet, and the anticodon recognition domain adopts the HGPT fold also found in HisRS, ProRS, and ThrRS.

The glycine binding site includes the conserved triad of glutamate residues described in previous studies [36-38, 62]. The flexible lid motif (residues 256–266) involved in recognition of the glycyl moiety includes the conserved ‘[AS]HY[AS]’ motif [90], with an ‘SHYS’ sequence occurring in *Mtb*GlyRS and ‘SHYA’ in *Mtr*GlyRS. The signature class-II ATP binding loop contains the conserved R_197_x…x[D/E]x…x[H/R]x…x[F_212_/Y] motif (*Mtb*GlyRS numbering) and helical segment with a highly conserved arginine residue (*Mtb*Arg331) within the previously defined motifs 2 and 3, respectively [36].

### Homodimeric properties and dimer interface remodeling

**Fig 3:**
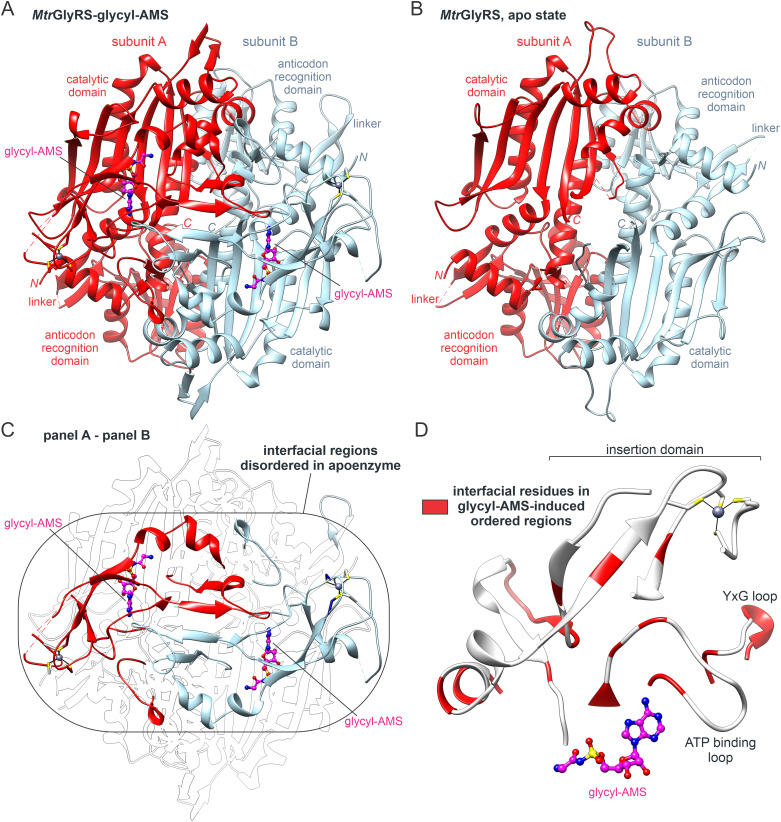
Stabilization of *Mtr*GlyRS dimer interface via complexation with glycyl-AMS. **(A)** Complex with glycyl-AMS displays more structurally ordered interface. **(B)** Apoenzyme crystallizes as dimer despite highly unstructured interface. **(C)** Regions that become ordered upon complexation with glycyl-AMS (ribbons, color labeled). **(D)** Residues within ordering regions forming intersubunit contacts (red).

Despite isolating the monomers during gel filtration, the two synthetases crystallized in the canonical dimeric configuration. The dimers are formed largely through the packing of the catalytic domains and display a domain arrangement similar to those observed in the *T. thermophilus* and human GlyRSs [36–38]. In the structures containing ligands, the dimerization elements visible in the electron density maps include the ATP binding loop, a YxG loop that inserts into a three-helix motif in the opposite chain, the lower edge of the insertion domain, and the β-hairpin that immediately follows the insertion domain (Figure 3A). The hairpin packs adjacent to the equivalent hairpin in the neighboring chain with the two-fold rotation axis relating the subunits located between them.

Remarkably, the interface visible in the electron density map of the *Mtr*GlyRS apoenzyme is quite limited (Fig 3B and C). The disordered sections include nearly 30 residues that form intersubunit contacts in the more ordered dimers, with greater than half being hydrophobic (Fig 3D). Interestingly, the theoretical dissociation free energies estimated for the glycyl-AMS-bound states exceed 24 kcal/mol whereas the value for the apoenzyme model having more missing structural elements is less than 1 kcal/mole [76].

### Glycyl-AMS and AMP binding sites

**Fig 4:**
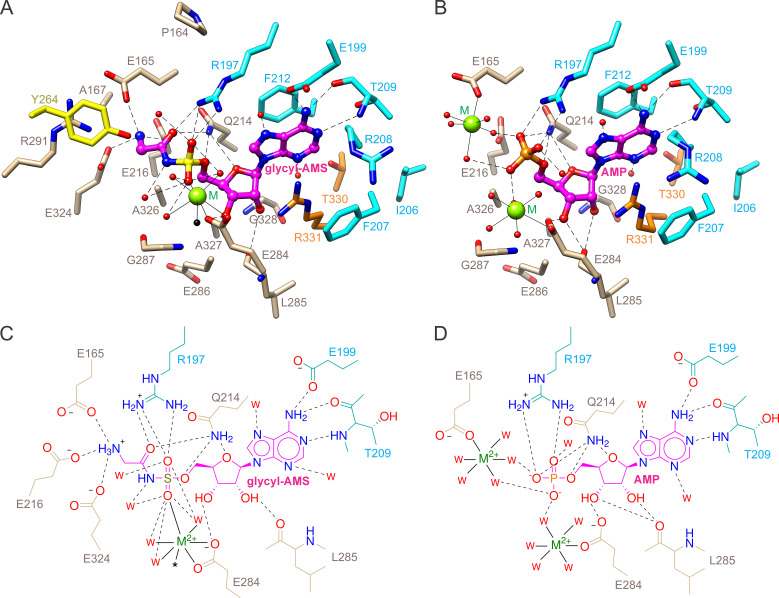
Glycyl-AMS and AMP binding sites in *Mtb*GlyRS crystals. **(A)** Glycyl-AMS. **(B)** AMP. The displayed residues reside within 5 Å of the ligands. The presence of two divalent metals in the complex with AMP is likely the result of high crystallant concentrations of magnesium and calcium. **(C,D)** Accompanying chemical drawings of polar contacts. Carbon atoms are colored as in Fig 2. Hydrogen and ionic bonds are drawn schematically using dashed lines, and coordination bonds involving the divalent cations using solid lines. Water molecules are depicted as red spheres or ’w’. The black sphere and asterisk indicate a buffer component.

In both the AMP and glycyl-AMS binding sites, the adenine moieties are interposed between Phe212 (*Mtb*GlyRS numbering) and Arg331 with the *N*_6_ amine engaging Glu199 and Thr209 through hydrogen bonding and *N*_1_ forming an additional hydrogen bond with Thr209 (Fig 4). The ribose moieties, which display 3’ endo puckering, likewise form similar contacts, the O2’ hydroxyl with nearby backbone sites, the O3’ hydroxyl with the carboxylate of Glu284 (a metal coordinating residue), and the O4’ and O5’ atoms with the side chain amide of Gln214. The sulfamoyl group binds the guanidinium of Arg197 and ligates the divalent metal via its two oxygens; the nitrogen does not interact with the protein but instead forms a hydrogen bond with an active site water (Fig 4A and C). The phosphoryl group of AMP aligns better and bonds ionically with the guanidinium of Arg197 (Fig 4B and D).

In the glycyl group binding site, the carbonyl oxygen forms hydrogen bonds with both the Arg197 and Gln214 side chains (Fig 4A and C). The amino group is held by the carboxylates of Glu216, Glu165, and Glu324. The configuration of the carboxylates, especially that of Glu324, together with the presence of the side chain of Ala326, impede the binding of pro S substituents, thus providing the molecular basis for the substrate specificity. Interestingly, Glu324 is oriented through a hydrogen bond with the more buried Glu218, which as noted previously for *Tt*GlyRS, must be substantially pKa-shifted [36].

### Active site remodeling

**Fig 5:**
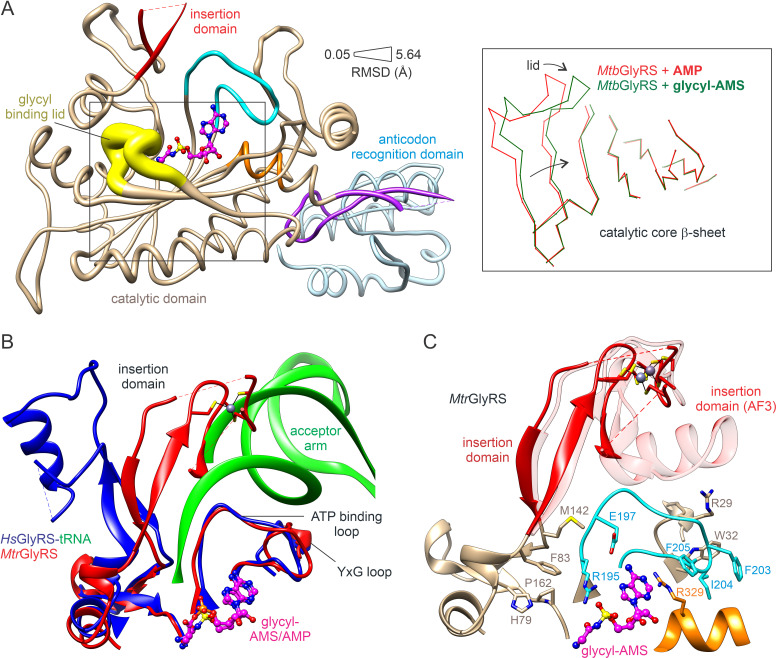
Active site remodeling. **(A)** Lid closure stabilization upon introduction of glycyl moiety in *Mtb*GlyRS. Magnitude of backbone conformational displacements are shown in tube representation. Tube thickness varies according to RMSD between *C*α atoms. Inset highlights twist in central β-sheet of catalytic domain that accompanies change in lid motif. **(B)** Glycyl-AMS binding orders ATP and YxG loops for tRNA binding in *Mtr*GlyRS (red). The loops adopt conformations resembling those observed in tRNA-bound human GlyRS (green and blue, PDB entry 5E6M [46]). **(C)** Active site stabilization induced by glycyl-AMS. Selected residues forming intersegmental contacts are displayed. An AlphaFold 3 model of the complete insertion domain is shown semi-transparently [91]. In panels A and C, the coloring schemes follow the convention used in Fig 2.

In transitioning from the AMP to glycyl-AMS bound states, the largest conformational change involves closure of the lid motif, with displacements having magnitudes as great as 5.6 Å that accompany a twist in the central sheet (Fig 5A). While the lid and glycyl-AMS lack close contacts, the hydroxyl group of Tyr264 is placed 3.6 Å from the electrophilic glycyl carbonyl carbon (Fig 4A and C).

Partial priming of the enzyme for tRNA binding is most evident in the series of *Mtr*GlyRS structures, which includes the apo state (Fig 5B). The ATP binding loop orders over the adenine moiety, with conserved Phe205 docking at Arg329, Glu197 and Thr207 at the adenine *N*_6_ amine, and Arg195 at the glycyl-sulfamoyl group. This conformational state introduces stabilizing interactions with the YxG loop, including electrostatic contacts between Gly201 and Arg29 and hydrophobic packing between Phe203 and Ile204 and Trp32 (Fig 5C).

The insertion domain also becomes increasingly ordered upon binding AMP and glycyl-AMS, as the lid structure becomes more organized. A helical insert between the knuckles of the zinc motif is largely disordered but likely situates at the tRNA acceptor arm binding site above ATP (Fig 5C). The two-stranded beta stem forming the base of the zinc motif head places Phe83 at the tRNA A76 binding site, where it packs between His79, Met142, Pro162, and Arg195. On the basis of the structure of tRNA-bound human GlyRS [46], a major conformational change must occur in this region to allow A76 to insert between and stack face-to-face with Phe83 and Arg195.

## Discussion

Structural genomics repositories are not unlike the Svalbard Global Seed Vault, safeguarding critical molecular resources against future biological crises. Providing the scientific community protein crystal structures from a vast array of infectious disease targets is anticipatory of a future rife with multi- and pan-resistant cellular pathogens, which has suddenly arrived at our doorstep. These structures can dramatically accelerate the design of targeted inhibitors, particularly against formidable pathogens like *M. tuberculosis*, where lengthy, multi-drug regimens already impose enormous healthcare costs. Because only 10–20% of structural genomics targets ultimately result in successful structure determination, these repositories help researchers sidestep negative results and prioritize efforts where atomic-level insight is available, allowing more efficient navigation of the early stages of drug discovery. Within this framework, the crystallographic analysis of *M. tuberculosis* GlyRS provides an additional promising resource that can be leveraged to expand our therapeutic toolchest against this persistent pathogen.

By linking tRNAs to their cognate amino acids, and in several cases editing misaminocacylations, the AaRSs are essential players in protein translation and hence have been intensely pursued as drug targets. However, careful characterization of the AaRSs for drug discovery typically benefits from structure determination of substrate-bound complexes, which often have more structured active sites that reflect natural conformational changes. The presence of substrates is also known to potentiate the binding of some inhibitors, as seen with cladosporin, agrocin derivative TM84, and halofuginone [92–94].

The active sites of AaRSs possess two class-specific motifs used for binding ATP [30, 95–97]. Several class II synthetases also utilize a molecular “lid” located on the opposite side of its core fold that adopts open and closed conformations, with the latter surrounding the amino acid substrate and aminoacyl moiety of the reaction intermediate. Flexibility in these regions is commonly observed, especially in the absence of ligands, and the conformational dynamics can be leveraged for inhibition. This is exemplified by the natural product borrelidin, which binds *Ec*ThrRS and human cytosolic ThrRS only in the open state of the lid motif [93, 98].

**Fig 6:**
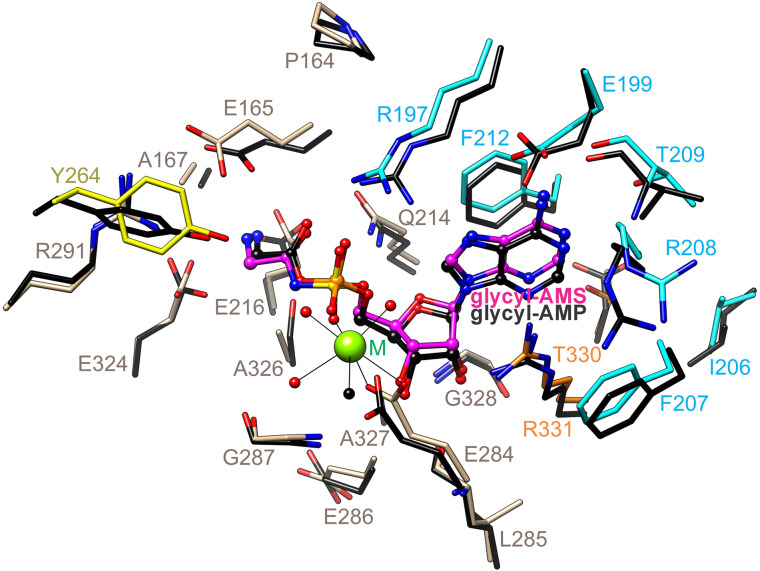
Glycyl-AMS binding site in *Mtb*GlyRS recapitulates glycyl-adenylate binding site in *Tt*GlyRS. Residues within 5 Å of the ligand are displayed in stick representation and are colored according to Fig 2B. The aligned residues in *Tt*GlyRS (PDB entry 1GGM, chain A [62]) are colored black.

The first class I and class II AaRS binding site architectures containing aminoacyl-adenylate analog inhibitors were characterized for glutaminyl-AMS-bound *E. coli* GlnRS [58] and seryl-AMS-bound *T. thermophilus* SerRS [57], respectively. The seryl-AMS was subsequently shown to adopt the same binding site architecture as that of seryl-adenylate, with the exception of a conformational change in an active site serine residue that interacts with the mainchain ester oxygen atom [99]. The present study describes the first crystal structures of bacterial class IIa GlyRSs bound to glycyl-AMS and magnesium, which closely recapitulate the binding site architecture of the glycyl-adenylate-bound *Tt*GlyRS (Fig 6) [62]. Notably, there are no protein contacts with the mainchain ester oxygen due to the use of a glycine substitution. The complexes with glycyl-AMS are also important for delineating the ordered state of the active sites, which instead show relaxed lid motif conformations or dynamics in complexes with AMP, or a much less well-defined active site structure in the case of the apo *Mtr*GlyRS.

By comparison with the Mycobacterial GlyRSs, the human GlyRS is a much larger enzyme, with isoforms of 739 and 685 amino acids [43, 44] that contain three insertions in the catalytic domain. The human enzyme further contains an *N*-terminal WHEP-TRS domain and a *C*-terminal extension absent in the Mycobacterial GlyRSs [37, 38, 46, 100] (S3A Fig in S1 File). The β-sheet of its catalytic domain is also expanded by two additional strands adjacent to strand six by the second insertion. Despite these differences, the catalytic cores and anticodon binding domains of the two orthologs are structurally highly conserved and overlay closely to ~1 Å RMSD (S3B Fig in S1 File). A key question is thus how easily can *Mtb*GlyRS inhibitors be developed that are selective over the human ortholog.

**Fig 7:**
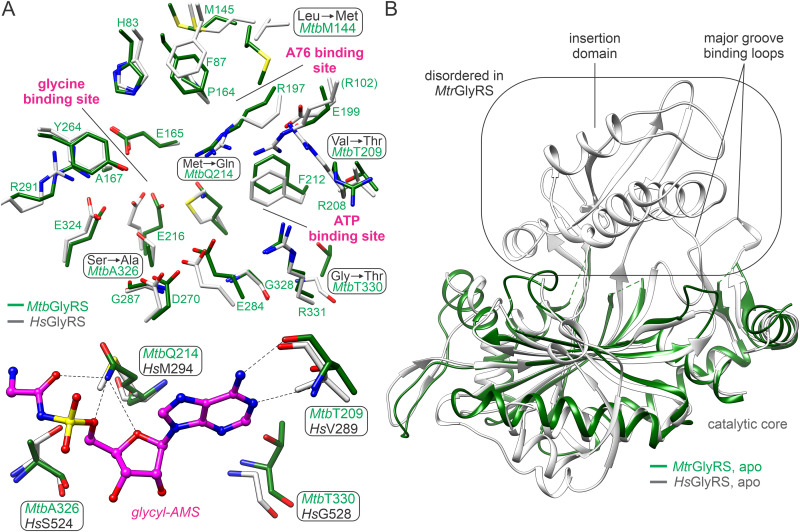
Structural and dynamics differences with human GlyRS catalytic domain. **(A)** Amino acid differences between *Mtb*GlyRS and *Hs*GlyRS in active site (top, circled) and glycyl-AMS subsite (bottom, circled). In the top panel, residues of *Hs*GlyRS that bind glycine, ATP, and tRNA A76 (grey, PDB entries 5E6M [46] and 2ZT7 [45]) are displayed together with the aligned residues of *Mtb*GlyRS (dark green). In the bottom panel, only residues proximal to the ligand are shown. **(B)** *Mtr*GlyRS and *Hs*GlyRS apoenzyme crystals exhibit contrasting degrees of structural order in their catalytic and insertion domains (PDB entry 2PME [38]).

Focusing on active site comparisons, five sites show amino acid replacements within the glycine, ATP, and A76 binding sites that might be targeted through inhibitor binding; four of these sites occur within the glycyl-AMS binding site (Fig 7A). As noted above, the glycyl *C*α is directed towards *Mtb*Ala326/*Mtr*Ala324, which likely plays a key role in amino acid specificity. In human GlyRS, the alanine is substituted with a serine (Ser524). The proximal *Mtb*Gln214, which forms multiple hydrogen bonds with glycyl-AMS, is substituted by a methionine (Met294). The glutamine residue is the most strongly conserved in bacterial α2-type GlyRSs among the five sites (S4 Fig in S1 File), and introduction of a hydrogen bonding donor and/or acceptor might be exploited for inhibitor selectivity. Near the *N*1-*C*2 atoms of the adenine moiety, two threonine residues, *Mtb*Thr209 and Thr330, are replaced by a valine and glycine (Val289 and Gly528), respectively. A perturbation of the local water structure in this region appears likely from related structures of human GlyRS.

Additional differences with the active site of human GlyRS could conceivably emerge upon ligand dissociation or when fewer ligand moieties are present. The pronounced disorder observed in the apo *Mtr*GlyRS crystal supports this notion: the insertion domain and two tRNA major groove recognition loops are absent in the electron density map (Fig 7B). These flexible features could offer strategic opportunities for achieving selective inhibitor binding on the basis of the “flexible target, rigid decoy” model [101].

## Supporting information

S1 File Phylogenetic clustering of Mycobacterial GlyRSs, electron density maps of co-crystallized ligands, additional structural comparisons with human GlyRS, and amino acid sequence alignment of α2-type bacterial GlyRS targets. (DOCX)
