# Supplementary material for: Crystal structures of Mycobacterium tuberculosis and Mycobacterium thermoresistibile glycyl-tRNA synthetases in various liganded states
Source: PLoS One. 2025 Jun 30;20(6):e0326500. doi: 10.1371/journal.pone.0326500 (PMC12208437; doi:10.1371/journal.pone.0326500)
Supplement: S1 File — (DOCX) [file pone.0326500.s001.docx]

**Supporting information**

**Crystal structures of *Mycobacterium tuberculosis* and *Mycobacterium thermoresistibile* glycyl-tRNA synthetases in various liganded states**

Michael K. Fenwick,^1,2,*^ Amy E. DeRocher,^1,2,*^ Justin K. Craig,^1,3^ Elizabeth K. Harmon,^1,3^ Steve Seibold,^1,4^ Lijun Liu,^1,4^ Kevin P. Battaile,^5^ Lynn K. Barrett,^1,3^ Wesley C. Van Voorhis,^1,3^ Isabelle Q. Phan,^1,2^ Bart L. Staker,^1,2^ Sandhya Subramanian,^1,2^ Scott Lovell,^1,4^ and Peter J. Myler^1,2,6,7,**^

^1^Seattle Structural Genomics Center for Infectious Disease, Seattle, Washington 98109, United States

^2^Center for Global Infectious Disease Research, Seattle Children’s Research Institute, Seattle, Washington 98109, United States

^3^Department of Medicine, Division of Allergy and Infectious Diseases, Center for Emerging and Re‐emerging Infectious Diseases (CERID), University of Washington, Seattle, Washington 98109, United States

^4^Protein Structure and X-ray Crystallography Laboratory, The University of Kansas, Lawrence, Kansas 66047, United States

^5^New York Structural Biology Center, X-ray Department, New York, New York 10027, United States

^6^Department of Biomedical Informatics and Medical Education, University of Washington, Seattle, Washington 98195, United States

^7^Department of Global Health, University of Washington, Seattle, Washington 98105, United States

*These authors contributed equally

**Correspondence: [Peter.Myler@seattlechildrens.org](mailto:Peter.Myler@seattlechildrens.org)

**
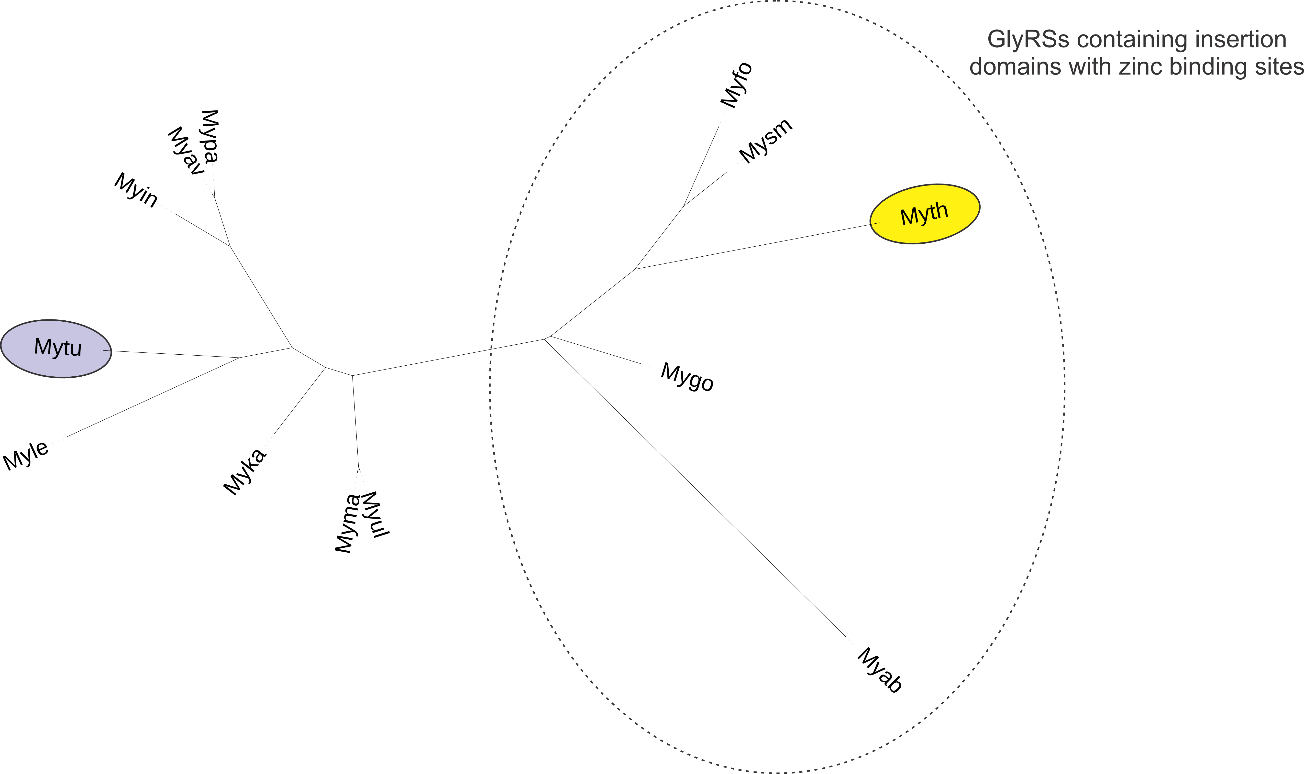
**

**Fig S1. Phylogenetic clustering of Mycobacterial GlyRSs having two types of insertion domains.** Orthologs containing a conserved zinc binding motif in their insertion domain are indicated. Amino acid sequences were aligned using Clustal omega [79] and a maximum likelihood tree was constructed with the aid of PhyML [102].The accession codes are as follows: *Mycobacterium abscessus*, B1MN75; *Mycobacterium avium*, A0A0H3A0F4; *Mycobacterium fortuitum*, A0A7Y4PB76; *Mycobacterium gordonae*, A0A0Q2X075; *Mycobacterium intracellulare*, X8A5M4; *Mycobacterium kansasii*, A0A1V3XNR2; *Mycobacterium leprae*, A0A0H3MYV6; *Mycobacterium marinum*, B2HLA7; *Mycobacterium paratuberculosis*, Q73Y21; *Mycobacterium smegmatis*, A0R0R9; *Mycobacterium thermoresistibile*, G7CIG9; *Mycobacterium tuberculosis*, P9WFV7; *Mycobacterium ulcerans*, A0PTS7.


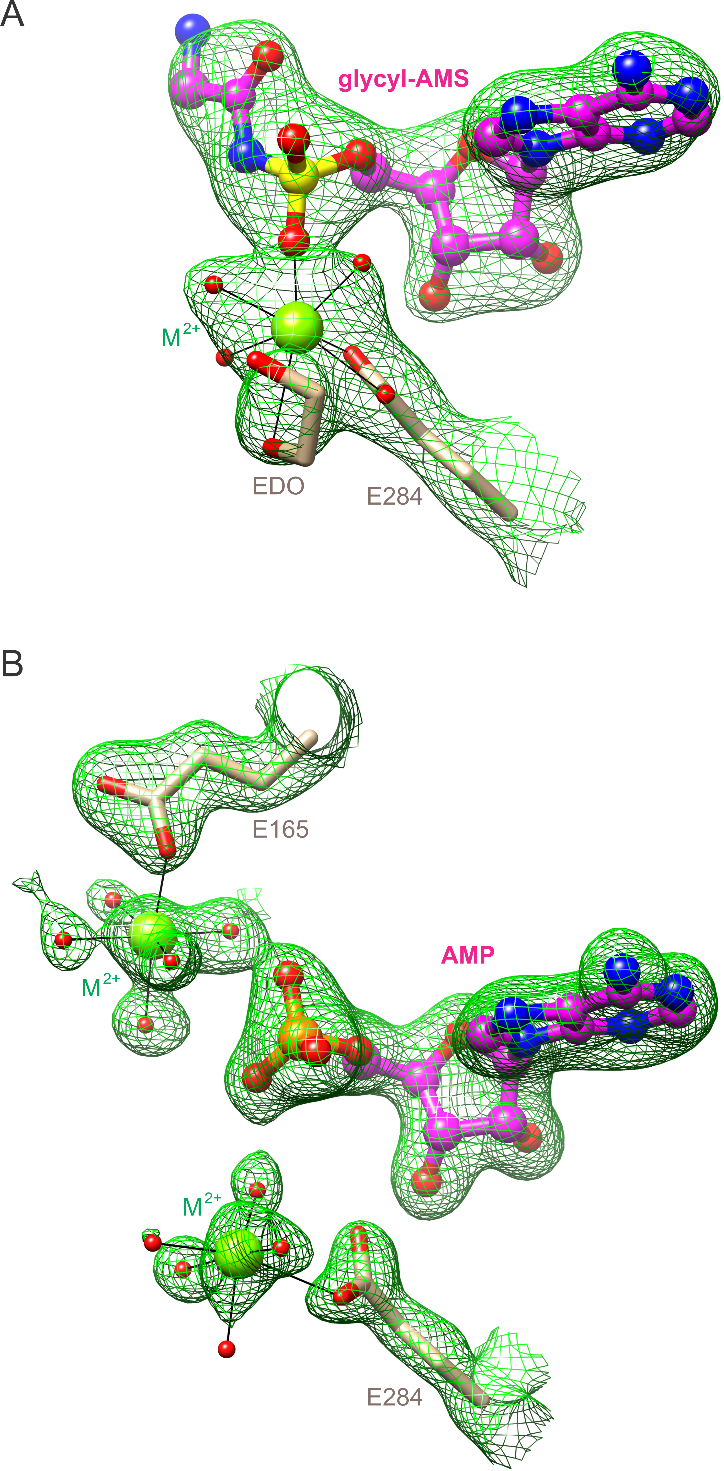


**Fig S2. 2*Fo*-*Fc* composite omit maps, displayed around ligands bound to *Mtb*GlyRS.** (A) Glycyl-AMS. (B) AMP. Coordination spheres of active site divalent metal ions are also displayed. Maps were generated using Phenix [74] and are contoured at approximately 1.5 σ using Coot [73] and Chimera [82].


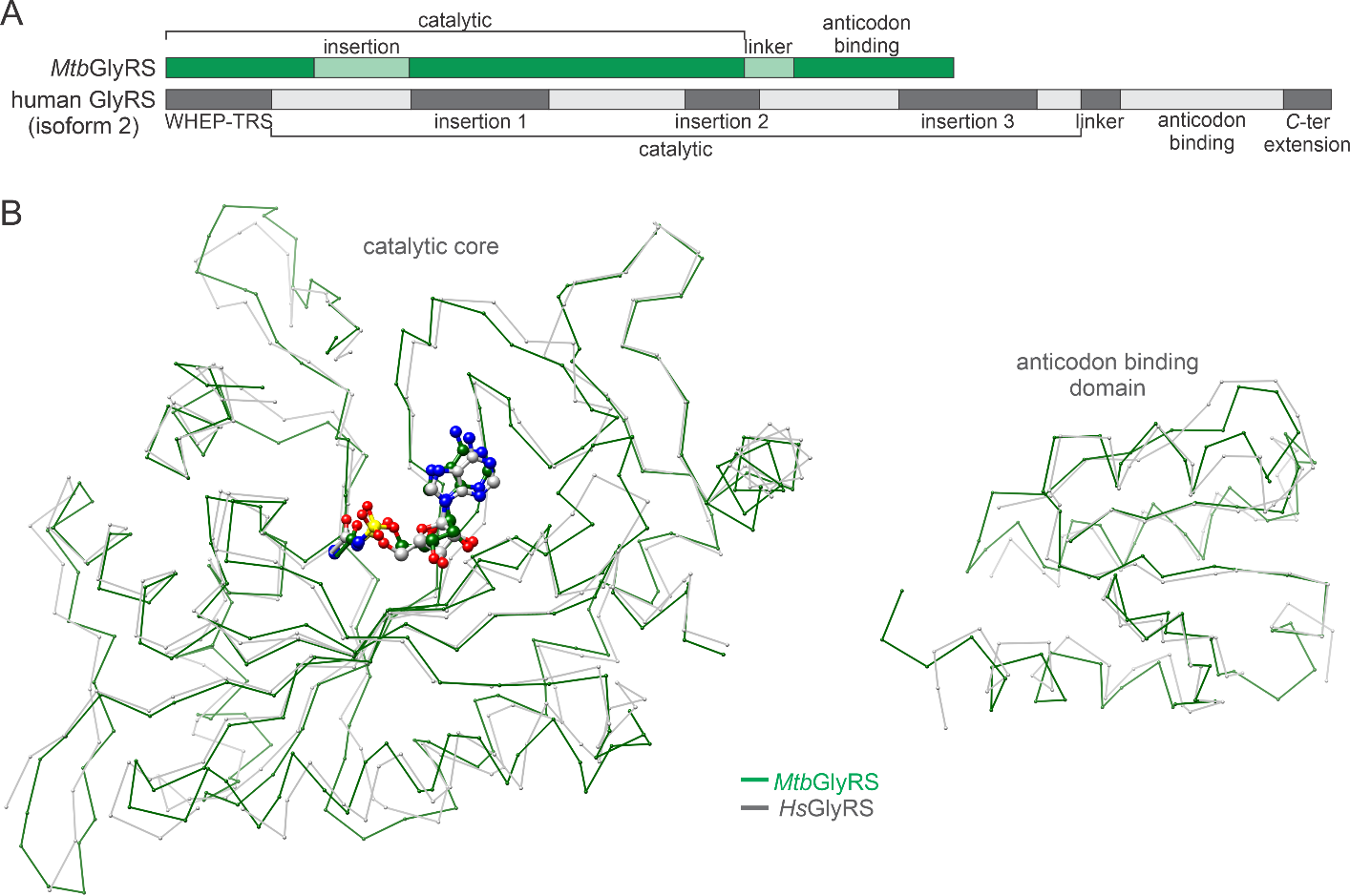


**Fig S3. Architectural similarities and differences between *M. tuberculosis* and human GlyRSs.** (A) Schematic representations of primary structures highlighting insertions and *N*- and *C*-terminal extensions in the human ortholog. (B) High structural homology in catalytic cores and anticodon binding domains (PDB entry 2ZT8 [45]).

10 20 30 40 50

. . . . .

*Mtu*GlyRS --MHHPVAPVIDTVVNLAKRRGFVYPSGEIYGGTKSAWDYGPLGVELKENIKRQWWRSVV

*Nbr*GlyRS ----MAPKSKVDTVANLAKRRGLVYPSGEIYGGTKSAWDYGPLGVELKENIKRQWWRSMV

*Gva*GlyRS -----MAVSKLDEVVSLAKRRGFVFPAGEIYGGTRSAWDYGPLGVALKDNIKREWWRYMV

*Cdi*GlyRS ----MAQNNVIDTVVNLCKRRGLVYPCGEIYGGTRSAWDYGPLGMELKENIKKQWWRTFV

*Lin*GlyRS MEKKESLDSSLKEIVSVCKRRGFVYPGSEIYGGLSNTFDYGPYGVELLQNLKQLWWKYFV

*Sau*GlyRS ------MAKDMDTIVSLAKHRGFVFPGSDIYGGLSNTWDYGPLGVELKNNVKKAWWQKFI

*Ban*GlyRS -------MYSMEQVVNLAKHRGFVFPGSEIYGGLANTWDYGPLGIELKNNVKKAWWKKFI

*Bfr*GlyRS ----MAQEDVFKKLVSHCKEYGFVFPSSDIYDGLGAVYDYGQMGVELKNNIKKYWWDSMV

*Pgi*GlyRS ---MAQQEDLFKKIVSHCKEYGFVFPSSEIYDGLAAVYDYAQYGSELKNNIKRYWWESMT

*Cca*GlyRS ---MINQEDFFKKVISHAKEYGYIFQSSEIYDGLSAVYDYGQNGVELKKNIREYWWKAMV

*Tpa*GlyRS ----------MEKIVGLCKRRGFVFPSSEIYGGQGGVWDYGPMGIALKNNIAHAWWQDMT

*Bbu*GlyRS -------MVRMEDIISLAKRKGFVFQSSEVYGGLSGAWDYGPLGVELKKNIKKEWWKSMV

*Mpn*GlyRS ----MAQVYSQEVYVQYLKRYGFVFQSSELYNGLANSWDFGPLGAVLKQQIKTALYNFFI

* * * * * * *

60 70 80 90 100 110

. . . . . .

*Mtu*GlyRS TGRDDVVGIDSSIILPREVWVASGHVDVFHDPLVESLITHKRYRADHLIEAYEAK-----

*Nbr*GlyRS TSREDIVGLDSSVILPRQVWVASGHVGVFNDPLVECLNCHHRFRQDHLQEAYALK-----

*Gva*GlyRS TTRGDVVGVDTSVILPSQVWVASGHVSVFNDPLIECLNCHKRHRADKLEESYAEK-----

*Cdi*GlyRS QARADVVGLDSSIILPRQVWVASGHVATFTDPLVESLHTHKRYRADHLIEAYEAK-----

*Lin*GlyRS HLREDIVGLDSSILLNPKVWEASGHVSNFNDPLIDCKNCKTRIRADKFLEDQKGEGFA--

*Sau*GlyRS TQSPFNVGIDAAILMNPKVWEASGHLNNFNDPMIDNKDSKIRYRADKLIEDYMQDVKGD-

*Ban*GlyRS QESPYNVGLDAAILMNPKTWIASGHVGNFNDPMIDCKKCKARHRADKLIED-ALDAKG--

*Bfr*GlyRS LLHENIVGIDSAIFMHPTIWKASGHVDAFNDPLIDNKDSKKRYRADVLIEDQLAKYDDKI

*Pgi*GlyRS LLHDNVVGIDSAIFMHPSIWKASGHVDAFNDPLIDNKDSKKRYRADVLVEDHLAKIDEKI

*Cca*GlyRS QMNENIVGIDAAIFMHPTTWKASGHVDAFNDPLIDNKDSKKRYRADVLVEDYVAKIEAKI

*Tpa*GlyRS RLHDHIVGLDAAILMHPNVWRTSGHVDHFSDPLVDCTVCKSRFRADQVAVPSAG------

*Bbu*GlyRS YLHENIVGLDSAIFMRPEIWRASGHVDGFSDSMVDCKDCKSRFRADFIDLSKN-------

*Mpn*GlyRS KNKRDVLLIDTPIILNEQIWKASGHLANFTDALVDCKSCKLRFRVDHLDEQIKS------

* * *** * * * * *

120

.

*Mtu*GlyRS ------------------------------------------------HGHPPPN--GLA

*Nbr*GlyRS ------------------------------------------------NKIDDPDTVSME

*Gva*GlyRS ------------------------------------------------HGDKMPEN-GLK

*Cdi*GlyRS ------------------------------------------------HGHAPEN--GLA

*Lin*GlyRS ---------------------------------------------TGLTLEKMNQVIKES

*Sau*GlyRS ----------------------------------------ENFIADGLSFEQMKKIIDDE

*Ban*GlyRS ----------------------------------------IEMVVDGLTFDQMADLMKEH

*Bfr*GlyRS NKEVAKAAKRFGEAFDEAQFRSTNGRVLEHQAKRDALHERFAKALNDNNLEELRQIIVDE

*Pgi*GlyRS AKEVVKAAKRFGEAFDEAQFRATNARVIEYQQKRDQIHERFATALNDNNLEEIRQIILDC

*Cca*GlyRS EKEVAKAEKRFGEAFDKEQFITTNARVVEYKKQADAILKRLAKSLENEDLADVKALIEEL

*Tpa*GlyRS ------------------------------------------------------------

*Bbu*GlyRS ------------------------------------------------------------

*Mpn*GlyRS -----------------------------------------------------ATQWNPK

130 140 150 160 170 180

. . . . . .

*Mtu*GlyRS DIRDPETGEPGQWTQPREFNMMLKTYLGPIETEEGLHYLRPETAQGIFVNFANVVTTARK

*Nbr*GlyRS LLACPNCGTVGKWTEPRDFNMMLKTYLGPIESEEGLHYLRPETAQGIFVNFANVMTTARK

*Gva*GlyRS DIACPDCGTRGNWTEPRDFNMMLRTHLGPVEDENSLHYLRPETAQGIFVDFKNVMTATRQ

*Cdi*GlyRS DVPDPETGQPGNWTEPQMFSGLMKTYLGPVDNEQGLHYLRPETAQGIFVNFKNVMTTARM

*Lin*GlyRS NFACPNCGQRGTFTEARDFNLMFKTSHGASAEDSLDIYLRPETAQGIFLNFKNVVSTTRR

*Sau*GlyRS GIVCPVSKT-ANWTEIRQFNLMFKTFQGVTEDSTNEIFLRPETAQGIFVNYKNVQRSMRK

*Ban*GlyRS EVKCPDCGS-EEFTEIRQFNLMFKTFQGVTESSTNEIFLRPETAQGIFVNFKNVQRSMRK

*Bfr*GlyRS EIACPISGTK-NWTEVRQFNLMFSTDMGSTADGSMKIYLRPETAQGIFVNYLNVQKTGRM

*Pgi*GlyRS EIVCPISGTR-NWTEVRQFNLMFATEMGSTADGAMKVYLRPETAQGIFVNFLNVQKTGRM

*Cca*GlyRS EIACPESGSK-NWTDVKQFNLMFGTKLGATADTAMDLYLRPETAQGIFVNFLNVQKTGRM

*Tpa*GlyRS -GPCPQCGG--ALTGVRNFNLMFSTHMGPTDERASLLYLRPETAQGIYVNYKNVLQTTRL

*Bbu*GlyRS -CPNCKVGN--NFTSPRSFNLMFKTHIGVVEDSSSEVYLRPETAQGIFVNFRNVLDSSRL

*Mpn*GlyRS QVNCPNCKAN-NWSEVRDFNLLFQTEIGVVNSEKRLVYLRPETAQGIFINFKQLLQLKKR

* * * *********

190 200 210 220 230 240

. . . . . .

*Mtu*GlyRS KPPFGIGQIGKSFRNEITPGNFIFRTREFEQMEMEFFVEPATAKEWHQYWIDNRLQWYID

*Nbr*GlyRS KPPFGIAQIGKSFRNEITPGNFIFRTREFEQMEMEFFVKPGEDAEWHKYWIETRFSWYTD

*Gva*GlyRS KPPFGIANIGKSFRNEITPGNFIFRTREFEQMEMEFFVEPGTDEAWHQYWIDTRVNWYVD

*Cdi*GlyRS KPPFGIAQVGKSFRNEITPGNFIFRTREFEQMEIEYFVPEELADQKFNEWVDDCWNWFVD

*Lin*GlyRS KIPFGIAQIGKSFRNEIMARQFVFRTREFEQMEMEFFCEPGTQKEWFSHWVNYCMNWLTE

*Sau*GlyRS KLPFGIGQIGKSFRNEITPGNFIFRTREFEQMELEFFCKPGEEIEWQNYWKTFASDWLTS

*Ban*GlyRS KLPFGIGQIGKSFRNEITPGNFTFRTREFEQMELEFFCKPGEDLEWFAFWRETCKNWLLS

*Bfr*GlyRS KVPFGIAQIGKAFRNEIVARQFIFRMREFEQMEMQFFVRPGSELEYFKKWKEIRLKWHKA

*Pgi*GlyRS KIPFGICQIGKAFRNEIVARQFIFRMREFEQMEMQFFVRPGEELQWFEKWKNLRMQWHRA

*Cca*GlyRS KIPFGIAQTGKAFRNEIVARQFIFRMREFEQMEMQFFIKPGTQKEWYEHWKEARLKWHLS

*Tpa*GlyRS KVPFGIAQIGKAFRNEIVTKNFIFRTCEFEQMEMQFFVRPAEDTHWFEYWCAQRWAFYQK

*Bbu*GlyRS KIPFGIAQVGKAFRNEIVTKNFIFRTCEFEQMEMQFFVHPKQIDEWFCYWQQNRMNFFIE

*Mpn*GlyRS PLPFGVAQFGKSFRNEVTPGNFLFRVREFEQFEMEWFCNPQASLSVFESQQQAIAHFLFK

*** ** **** * ** **** * *

250 260 270 280 290 300

. . . . . .

*Mtu*GlyRS -LGIRRENLRLWEHPKDKLSHYSDRTVDIEYKFGFMGNPWGELEGVANRTDFDLSTHARH

*Nbr*GlyRS -LGINPDNLRLYEHPKEKLSHYSAGTTDIEYRFGFQGNEWGELEGVANRTDYDLKTHSEH

*Gva*GlyRS -LGVKPENLRMYEHPKEKLSHYSKRTVDIEYKFGFQGSDWGELEGIANRTDYDLSAHAKH

*Cdi*GlyRS -LGINPDNMRRFDVPEEDRAHYSKGTIDMEYKFGFQGNPWGELMGVANRTDYDLGCHIKE

*Lin*GlyRS QVGIKKENLRVREHEKEELSFYSEGTSDIEFKYNFG---WGELWGIASRTDYDLNQHQKF

*Sau*GlyRS -LNMSSENMRLRDHDEDELSHYSNATTDIEYKFPFG---WGELWGIASRTDFDLRKHAEH

*Ban*GlyRS -LGMTEESMRLRDHGEEELSHYSNATTDIEFKFPFG---WGELWGVASRTDFDLKRHMEH

*Bfr*GlyRS ---LGFGDDHYRFHDHDKLAHYANAATDIEFLMPFG---FKEVEGIHSRTNFDLSQHEKF

*Pgi*GlyRS ---LGFGDDKYRFHDHDKLAHYANAATDIEFEMPFG---FKEVEGIHSRTDFDLSRHEEF

*Cca*GlyRS ---LGMGSENYRFHDHEKLAHYADAACDIEFRFPFG---FKELEGIHSRTDFDLGNHEKY

*Tpa*GlyRS -YGVRMNHMRWRTHAAHELAHYARAACDIEYAFPMG---FRELEGVHNRGDFDLTRHAQH

*Bbu*GlyRS TLKISPDRLRFKAHDSTQLAHYAKAAFDIEYEFPFG---FQEVEGIHNRGNYDLTQHAKF

*Mpn*GlyRS VLQLNPALVKQYEYDKNELAHYANKTVDFLFQFPHG---LRELWGLADRGTFDLEQHQKY

* * * * * ** *

310 320 330 340 350

. . . . .

*Mtu*GlyRS SGVDLSFYDQIND-----VRYTPYVIEPAAGLTRSFMAFLIDAYTEDEAPNTKGG-----

*Nbr*GlyRS SGTELSFFDQTTN-----ERYIPYVIEPAAGLTRSLMAFLVDAYAEDEAPNAKGG-----

*Gva*GlyRS SGEDLSYFNQATG-----EKYVPYVIEPAAGLTRSLMAFLVDAYDVDEAPNTKGG-----

*Cdi*GlyRS SGEDLSYFDQTTG-----ERYVPWVIEPSFGLTRALMAFLVDAYCEDEAPNAKGG-----

*Lin*GlyRS SGEDLKYQDQVQN-----KKYVPFVVEPALGVNRLFLAVVTDAYEEEKLPDGE-------

*Sau*GlyRS SGEDFRYHDPETN-----EKYIPYCIEPSLGADRVTLAFLCDAYDEE---GVEGS-----

*Ban*GlyRS SNEDFNYIDPQTN-----ERYVPYCIEPSLGADRVTLAFLCDAYEEE---QLE-------

*Bfr*GlyRS SGKSIKYFDPELN-----ESYTPYVIETSIGVDRMFLSIMSAAYCEEQLEN---------

*Pgi*GlyRS SGKKLQYFDPEQN-----KSYVPYVVETSIGLDRMFLTILFGSYCEEETSN---------

*Cca*GlyRS SGKKLQYFDPEEN-----KSYVPYVLETSIGLDRMFLAVLSNSLQEETLEG---------

*Tpa*GlyRS SGKDLCYVDPDPNLDAAARRYVPCVVETSAGLTRCVLMFLCDAYTEEYVQAPNVAFSETT

*Bbu*GlyRS SNKPKVFEYHDL---LTKEKYVPYVIETSAGLTRSVLMTLCDAYSEEELSD---------

*Mpn*GlyRS AKKPLDFFDGENN-----EHFIPAVVEPSVGIERLFYALIVSSYQQEQLEG---------

* * * *

360 370 380 390 400

. . . . .

*Mtu*GlyRS -----------MDKRTVLRLDPRLAPVKAAVLPLSRHADLSPKARDLGAELRKC-WNIDF

*Nbr*GlyRS -----------VDTRTVLRLDRRLSPVKAAVLPLSRNADLTPKAKDLAAQLRRN-WNVEF

*Gva*GlyRS -----------VDKRTVLRLDPRLAPVKAAVLPLSKKPELQAVAQDLASDLRQHDWVIDY

*Cdi*GlyRS -----------VDKRVVLKLDPRLAPVKVAVLPLSKKDTLTPVAEEVAAKLRQF-WNVDY

*Lin*GlyRS -------------TRTVLRFSPKIAPVKAAIFPLMKKDGLPEKSREIFADLSKL-GNIEY

*Sau*GlyRS -----------KDARTVLHFHPALAPYKAAILPLSKK--LSGEAIKIFEQLSSK-FSIDF

*Ban*GlyRS -----------NDSRTVLRFHPALAPYKAAILPLSKK--LSEGATEVFAELAKD-FMVDF

*Bfr*GlyRS -----------GESRVVLKLPAALAPVKLAVMPLVKKDGLPEKAREIIDNLKFH-FHCQY

*Pgi*GlyRS -----------GEMRVVLKLPAALAPVKLAVLPLVRKDGLDAKAREIVHDLRFD-FACQY

*Cca*GlyRS -----------GDTRTVLRLPFVLAPTKVAVLPLLKKDGLPEIAREIIDELKFD-FSVSY

*Tpa*GlyRS QTADQEGAARTGEMRIVLRLHPALSPTTVAFLPLVKKDGLVDLARAVRDELRED-FACDF

*Bbu*GlyRS -----------GDKRIVLRLHPKLAPYKIAIFPLVKKVELTEIARRIYMELCDD-FHIFY

*Mpn*GlyRS ------------EMREVLRLPFHLCPEQIVVLPLVNK--LKETAQTLFEALSQTHWRIGF

* ** * ** * *

410 420 430 440 450 460

. . . . . .

*Mtu*GlyRS DDAGAIGRRYRRQDEVGTPFCVTVDFDSLQDNAVTVRERDAMTQDRVAMSSVADYLAVRL

*Nbr*GlyRS DDAGAIGRRYRRQDEIGTPFCITVDFDTLEDQAVTIRERDSMAQERIALDKVEGYLAQHL

*Gva*GlyRS DESGAIGRRYRREDEIGTPLCVTVDFDTLEDHAVTIRERDTMQQERVSLDKVADYVASRI

*Cdi*GlyRS DTSGAIGRRYRRQDEIGTPFCVTVDFDTLEDNAVTVRERDTMTQERVALADLQSYLAARL

*Lin*GlyRS DDGGAIGKRYRRQDEIGTPFCITVDYDTLKDDTVTVRERDSMSQERIAVNQLKNWLFERL

*Sau*GlyRS DESQSIGKRYRRQDEIGTPYCVTFDFDSLEDNQVTVRDRDSMEQVRMPISELEAFLTEKT

*Ban*GlyRS DETGSIGKRYRRQDEIGTPFCITYDFDSVEDKAVTVRDRDTMEQVRMPISELKGFLEKKI

*Bfr*GlyRS DEKDSIGKRYRRQDAIGTPYCVTVDHQTLEDNCVTLRNRDTMEQERVAISELNNIIADRV

*Pgi*GlyRS DEKDSIGKRYRRQDAIGTPFCITVDHQSLEDNTVTIRYRDTMEQERVEISRLNAIISEYV

*Cca*GlyRS DEKDAVGRRYRRQDAAGTPFCITVDHQTKEDQTVTLRHRDTMEQIRIPISELRSVISKEV

*Tpa*GlyRS DAAGAIGKRYRRQDEVGTPFCVTVDYQSKEDDTVTVRLRDSMAQRRVSRAFLAEFLRTEI

*Bbu*GlyRS DDSGTIGKRYRRQDEIGTPYCVTIDYNTIEDETVTVRERNSMTQKRIFINDLYSYIKTEI

*Mpn*GlyRS ESAGSIGKRYRKADAIGTKFAITFDFESLEDQAVTIRERDSLKQVRVPIKELKAWFAQHD

* *** * ** * * * ** * * * *

*Mtu*GlyRS KGS--------------------------------------------

*Nbr*GlyRS LGS--------------------------------------------

*Gva*GlyRS NEKRVKYPQGPVEIVGTRAADGGVDVSKENGVDESQPVKIAQAGGLY

*Cdi*GlyRS IGC--------------------------------------------

*Lin*GlyRS -----------------------------------------------

*Sau*GlyRS KF---------------------------------------------

*Ban*GlyRS QF---------------------------------------------

*Bfr*GlyRS SITSLLKTIQ-------------------------------------

*Pgi*GlyRS SLKSLLKKIDL------------------------------------

*Cca*GlyRS DMRNWLKKQF-------------------------------------

*Tpa*GlyRS KHYRRP-----------------------------------------

*Bbu*GlyRS LNYKEDFNK--------------------------------------

*Mpn*GlyRS DQSH-------------------------------------------

**Fig S4. Sequence conservation of *α*2-type bacterial GlyRSs**. Multiple sequence alignment of bacterial GlyRS targets from Clustal X2.1 [78]. Amino acids in the substrate binding site that vary between *Mycobacterium* and Human forms are highlighted green. Conserved sites are marked with asterisks. Accession codes are as follows: P9WFV7, *Mycobacterium tuberculosis*; K0EWF2, *Nocardia brasiliensis*; E3D949, *Gardnerella vaginalis*; Q6NG25, *Corynebacterium diphtheriae*; Q8F6C0, *Leptospira interrogans*; P99129, *Staphylococcus aureus*; Q81XT3, *Bacillus anthracis*; Q5LIA2, *Bacteroides fragilis*; B2RH83, *Porphyromonas gingivalis*; F9YSW0, *Capnocytophaga canimorsus*; O83678, *Treponema pallidum*; O51344, *Borrelia burgdorferi;* P75425, *Mycoplasma pneumoniae*.

**References**

102. Guindon S, Dufayard JF, Lefort V, Anisimova M, Hordijk W, Gascuel O. New algorithms and methods to estimate maximum-likelihood phylogenies: assessing the performance of PhyML 3.0. Syst Biol. 2010;59(3):307–21. Epub 2010/06/09. doi: 10.1093/sysbio/syq010. PubMed PMID: 20525638.
